# Promoting Effects of a Single *Rhodopseudomonas palustris* Inoculant on Plant Growth by *Brassica rapa chinensis* under Low Fertilizer Input

**DOI:** 10.1264/jsme2.ME14056

**Published:** 2014-08-12

**Authors:** Wai-Tak Wong, Ching-Han Tseng, Shu-Hua Hsu, Huu-Sheng Lur, Chia-Wei Mo, Chu-Ning Huang, Shu-Chiung Hsu, Kung-Ta Lee, Chi-Te Liu

**Affiliations:** 1 Department of Agronomy, National Taiwan University, No. 1, Sec. 4, Roosevelt Road, Taipei 106, Taiwan; 2 Institute of Biotechnology, National Taiwan University, R412., No. 81, Chang-Xing St., Taipei, 106, Taiwan; 3 Department of Plant Pathology and Microbiology, National Taiwan University, No. 1, Sec. 4, Roosevelt Road, Taipei 106, Taiwan; 4 Great Victory Chemical Industry Co., Ltd. 14F-1, NO. 206, Sec. 2 Nanjing E. Rd., Taipei 104, Taiwan; 5 Department of Biochemical Science and Technology, National Taiwan University, 1 Roosevelt Rd. Sec. 4, Taipei 106, Taiwan; 6 Agricultural Biotechnology Research Center, Academia Sinica, No. 128 Sec. 2, Academia Rd., Nankang, Taipei, 115, Taiwan

**Keywords:** PGPR, purple non-sulfur phototrophic bacteria, *Rhodopseudomonas palustris*, biofertilizer, integrated fertilization

## Abstract

Several *Rhodopseudomonas palustris* strains have been isolated from rice paddy fields in Taiwan by combining the Winogradsky column method and molecular marker detection. These isolates were initially screened by employing seed germination and seedling vigor assays to evaluate their potential as inoculants. To fulfill the demand in the present farming system for reducing the application of chemical fertilizers, we assessed the plant growth-promoting effects of the *R. palustris* YSC3, YSC4, and PS3 inoculants on *Brassica rapa chinensis* (Chinese cabbage) cultivated under a half quantity of fertilizer. The results obtained showed that supplementation with approximately 4.0×10^6^ CFU g^−1^ soil of the PS3 inoculant at half the amount of fertilizer consistently produced the same plant growth potential as 100% fertility, and also increased the nitrogen use efficiency of the applied fertilizer nutrients. Furthermore, we noted that the plant growth-promotion rate elicited by PS3 was markedly higher with old seeds than with new seeds, suggesting it has the potential to boost the development of seedlings that were germinated from carry-over seeds of poor quality. These beneficial traits suggest that the PS3 isolate may serve as a potential PGPR inoculant for integrated nutrient management in agriculture.

The application of excess amounts of chemical fertilizers in current farming systems has led to environmental hazards, such as nitrate contamination in ground water, the surface runoff of phosphorus, and eutrophication of aquatic ecosystems (13, 16). In recent years, the pursuit of quality, fresh, non-toxic, and safe products has become a trend in global agricultural production. One promising method to reduce the negative environmental effects caused by agricultural chemicals is the application of plant growth-promoting rhizobacteria (PGPR) as microbial inoculants in farming. PGPR can improve soil fertility, enhance plant nutrition availability and uptake, and support the health of plants (4, 8, 9, 35, 41, 42, 53, 65). The efficacy of PGPR has been validated in various greenhouse and field assessments with numerous plant species, and there have been many commercial inoculants that have been used in practical agricultural production (10, 41, 47, 59). Several studies recently demonstrated that the combined use of PGPR in the form of biofertilizers and reduction in the amount of chemical fertilizers applied could sustain soil fertility and crop yield (3, 4, 17, 36, 58). This integrated fertilization has been regarded as a promising method for the rational use of fertilizers to make agriculture more sustainable and productive.

*Rhodopseudomonas palustris* is one of the phototrophic purple non-sulfur bacteria (PNSB) that belong to the class α-*proteobacteria* (32). This bacterium is widely distributed in various aquatic ecosystems as well as in sediments, moist soils, natural wetlands, and paddy fields (29, 48, 52). *R. palustris* is able to grow under photoautotrophic, photoheterotrophic, chemoautotrophic, and chemoheterotrophic conditions and may play an important role in the nutrient cycles of natural environments (31, 38, 56). Due to its extraordinary metabolic versatility, *R. palustris* has been extensively used in industries for bioremediation and sewage treatment (27, 28, 34). Regarding the agricultural applications of this species, a mixed-strain inoculant containing *R. palustris* and other bacteria, such as lactic acid bacteria (*e.g.*, *Lactobacillus plantarum* and *L. casei*,), actinomycetes (*Streptomyces* spp.), and yeasts (*Saccharomyces* spp.), was previously shown to be useful for crops (21, 33, 67). However, the contribution of the individual *R. palustris* strain is not clear because the beneficial effects of the inoculant were mainly described in the presence of microbial consortia. In a previous study, Harada *et al.* (25) inoculated a *R. palustris* KN122 isolate to rice (*Oryza sativa*) seedlings with basal fertilizers (P and K), and the grain yield was 9% higher in the inoculated pots than in the non-inoculated controls. These findings suggested that *R. palustris* has the potential to act as single-strain PGPR inoculant.

Small-scale farmers in Taiwan mainly apply various fertilizers (organic, mineral, straight, and compound) at levels higher than the official nutrient recommendations to produce a wide variety of crops (tropical, temperate, traditional, innovative, staple, and high-value) in a range of climatic and geographical conditions (40). A high rate of N fertilizer is typically applied continuously to sustain proper growth and maximal high yield, especially for the successive cultivation of vegetable crops in the same fields (39). To promote low chemical input and ensure sustainable crop production, the administrative department of the government (Executive Yuan) has proposed a 50% reduction in the use of agrochemicals as a long-term agricultural policy since 2006. Therefore, the objective of this study was to identify promising PGPR inoculants that can preserve adequate soil fertility and crop productivity while supplementing the crops with half of the conventional fertilizer dosage. We isolated and characterized three *R. palustris* strains (PS3, YSC3, and YSC4) from Taiwanese rice paddy soils and evaluated their plant growth-promoting traits with a 50% reduction in fertilizer.

## Materials and Methods

### Isolation of purple non-sulfur phototrophic bacteria

A combination of the conventional enrichment and molecular marker detection methods was developed to isolate the purple non-sulfur phototrophic bacteria (PNSB). Bulk soil samples were gathered from two rice paddy fields: one at National Taiwan University (Taipei City, Taiwan) and another at the Hualien District Agricultural Research and Extension Station in Yilan County, Taiwan. Soil samples were transferred into Winogradsky columns to provide an enrichment culture for PNSB, as previously described (64). When the visual development of red growth zones in the enrichments occurred in a few days, the red plaques were collected for anaerobic incubation in a modified van Niel medium (hereafter designated as PNSB medium) (12). Incubation was conducted at 30°C under overhead fluorescent lamps (*ca.* 30 μmol photons m^−2^ s^−1^) in an incubator. When small red colonies appeared on the medium, a specific primer set was used to target the *pufM* gene, encoding a protein for the M subunit of the photosynthetic reaction center in PNSB (Table 1) (2). The PCR amplification product was expected to be 229 bp. The pure strains were then isolated by streaking agar plates. One of the bacterial isolates, PS3, which had beneficial effects on plant growth, was saved at the Bioresource Collection and Research Center (BCRC) in Taiwan with an accession number (BCRC910564). A representative type strain of *R. palustris* (BCRC 16408^T^ = ATCC 17001^T^) was purchased from BCRC.

### Seed germination and seedling vigor tests

To pre-evaluate the potential for plant growth promotion of the PNSB isolates, we performed germination and seedling vigor tests with *Brassica rapa chinensis* (non-heading Chinese cabbage) seeds. *B. rapa chinensis* is an annual crop that can grow in the cool season and is a very popular vegetable crop in Taiwan. Two lots of seeds were purchased sequentially from Known-You Seed Co., Ltd. in Taiwan. One lot was produced in March 2011, and the other was produced in May 2013. The two seed lots were preserved in the refrigerator before testing. The old lot seeds were used between June 2011 and July 2013. The new lot seeds were used since May 2013. The seeds were surface-sterilized with 1% sodium hypochlorite for 10 min. The seeds were inoculated by soaking in the respective *R. palustris* strain containing at least 10^8^ CFU mL^−1^ for 15 min. The seeds of the control treatments were soaked in sterile water or culture medium. Ten of the inoculated seeds were placed on one wateragar plate (1.5% sterile agar) with three replicates for each treatment in the dark at 30°C as described by (51). After being incubated for 3 d, the germinated seeds were counted. We measured the lengths of the roots and shoots of individual seedlings at the end of the 3-d incubation in order to determine seedling vigor. The vigor index was calculated using the following formula: Vigor index = (mean root length+mean shoot length) × (% germination), as described previously (1). The above experiments were performed independently at least 3 times.

### 16S ribosomal RNA, *puf* gene, and ITS region sequencing and phylogenetic analyses

The genomic DNA of bacteria was isolated from liquid cultures grown in PNSB broth using a Genomic DNA Mini Kit (Geneaid Biotech Ltd., Taiwan) according to the manufacturer’s instructions. The 16S rDNA fragment, 16S-23S rDNA ITS region, and *puf* gene from each isolate were amplified using the primers shown in Table 1. Gene sequences were determined by the Center for Biotechnology (National Taiwan University) and compared to sequences in the GenBank database using the Basic Local Alignment Search Tool (BLAST) (5). Sequence alignment and analysis of similarities between the genes were performed using the CLUSTALW program (62). Evolutionary distances were calculated, and the phylogenetic trees were constructed using the neighbor-joining (NJ) method (54). The topology of the trees was evaluated by bootstrapping with 1,000 resamplings (22). The phylogenetic trees were drawn using the MEGA5 program (24).

### Physiological characterization of bacterial isolates

The morphology and Gram staining of the vegetative cells was observed by optical microscopy (BH-51, Olympus, Tokyo, Japan), and single colonies were observed by stereomicroscopy (VEM-100, Optima, Taipei, Taiwan). Growth at various temperatures (5–50°C), pH ranges (pH 3.0–10.0), and NaCl concentrations (0–5% [w/v]) were investigated in the PNSB broth. The viability of the bacteria under different growth conditions was determined by measuring turbidity (OD_600_) and streak plating the cultures.

### Modification of the API 50CH test

The utilization of carbohydrates by each isolate was determined using a modified API 50CH method developed in this study. The original API 50 CH Medium (BioMérieux, France) containing phenol red (a pH indicator) was replaced with an adjusted L2 medium (61) excluding D-L sodium lactate and adding 7.6 mM (NH_4_)_2_SO_4_ with 0.01% (w/v) resazurin dye (Sigma-Aldrich, St Louis, MO, USA). When resazurin is reduced to resorufin by bacterial respiration, a blue to pink color change can be observed. Resorufin is further reduced to hydroresorufin, which lacks color. Accordingly, resazurin has been used as a surrogate indicator of bacterial growth (55). All test strips were inoculated with cell suspensions grown in PNSB broth. The API 50CH strips were used according to the manufacturer’s instructions. This improved API 50CH assay was designated as the API 50CH+LR method. The utilization reactions were observed for 24 and 48 h. All the tests were performed in triplicate.

### API ZYM tests

Extracellular enzyme activities were determined using the API ZYM system (BioMérieux) under aerobic conditions. The API ZYM strip was read after a 4-h incubation at 30°C. The tests were performed according to the manufacturer’s instructions. The intensity of each enzymatic reaction was semi-quantitatively assessed using an API ZYM color chart. All the tests were performed in triplicate.

### Acetylene-reduction assay of nitrogen-fixation activity

The nitrogen-fixing ability of the isolates was tested using an acetylene-reduction assay (ARA) (61) with some modifications. The cultures grown in PNSB broth were centrifuged and resuspended in L2 medium to an optical density at 600 nm (OD_600_) of 0.1. These cultures (50 mL) were incubated in 250 mL Erlenmeyer flasks sealed with sterile rubber septa. The gas phase in the flasks was replaced with N_2_ gas containing 3% air (~0.6% O_2_) and 10% C_2_H_4_. The flasks were incubated at 30°C, shaken at 200 rpm, and uniformly illuminated by overhead fluorescent lamps (*ca.* 30 μmol photons m^−2^ s^−1^) in an incubator. To measure the hourly rate of ARA, gas was sampled from the headspace of the flask at intervals, and the concentrations of acetylene and ethylene were measured using a gas chromatograph (Hitachi G-3000, Tokyo, Japan) equipped with a HayeSep T80/100 packed column (Supelco Inc., Bellefonte, PA, USA) and flame ionization detector (FID). The OD_600_ of each sample was measured immediately following gas sampling.

### Determination of indole-3-acetic acid

The production of indole-3-acetic acid (IAA) by bacteria in the culture supernatants was determined using Salkowski’s colorimetric method (50) with some modifications. Bacterial supernatant (100 μL) was mixed with the same volume of Salkowski’s reagent (150 mL of concentrated H_2_SO_4_ and 7.5 mL of 0.5 M FeCl^3^ L ^−1^) and incubated in a 96-well plate at room temperature for 30 min. The development of a pink color in the mixture indicated that indoles were synthesized. The absorbance of the samples was measured at 530 nm using a microplate reader (Victor3^TM^ 1420 Multilabel Counter; PerkinElmer Inc., Waltham, MA, USA). The quantity of indoles was determined by comparing with a standard curve prepared with a known concentration of IAA.

### Preparation of inoculants

Individual *R. palustris* isolates were grown in 500 mL Erlenmeyer flasks at 30°C, 180 rpm, with 150 mL of PNSB broth. Growth was determined by optical density at 600 nm, and bacteria were harvested around OD_600_=1.0. Colony-forming units (CFU) mL^−1^ were determined using the standard serial dilution method by plating on PNSB agar medium. The broth containing 10^8^–10^9^ CFU mL^−1^ of the *R. palustris* isolate was used for pot trials.

### Pot experiments to evaluate the plant-growth promotion of Chinese cabbage

The pot experiments described hereinafter were conducted using the two lots of Chinese cabbage seeds described above. Chinese cabbage seedlings were sown in soil-filled pots (containing approximately 300 g of soil) and grown in the Phytotron (Agricultural Experimental Station, National Taiwan University, Taipei, Taiwan) with natural sunlight at 25/20°C day/night and 80 (±5) % relative humidity. The pot experiments were conducted in a randomized complete block design with the following four treatments: (a) 0% CF, no chemical fertilizer (CF) or inoculant; (b) 100% CF, full amount of fertilizer alone; (c) 50% CF, half amount of fertilizer alone; and (d) 50% CF + respective *R. palustris* inoculant, inoculation of the bacterial strain (PS3, YSC3, YSC4, or the type strain BCRC16408^T^) with half of the standard amount of fertilizer. Each treatment had 20 replicates. The chemical fertilizer had an N : P : K ratio of 14 : 15 : 10 (Sinon Chemical Industry Co., Ltd., Taiwan). The 100% fertilizer amount was applied in accordance with the manufacturer’s recommendations (0.1 g pot^−1^, equivalent to N: P_2_O_5_ : K_2_O = 88 : 95: 63 [kg ha^−1^]), and the plants were fertilized once a week. The dosage of the microbial inoculants was based on our preliminary findings (data not shown). The concentration of each inoculant was adjusted to 1.5 × 10^8^ CFU mL^−1^, and 8 mL of a live or 65°C heat-killed bacterial suspension was applied to the surface soil of each pot (the total amount of the inoculant was 1.2×10^9^ CFU, equivalent to 4.0×10^6^ CFU g^−1^ soil). The plants were harvested from the pot 4 weeks after planting, and the plant fresh and dry weights were recorded.

The population of respective *R. palustris* inoculants in rhizosphere soil after 4 weeks of cultivation was analyzed using the most probable number (MPN) method described by Harada *et al.* (25). The remaining N in the soil was assessed using the Reflectoquant System (Merck Millipore, Darmstadt, Germany) to determine the NH_4_^+^ or NO_3_^−^ ion concentration according to the procedures described previously (57).

Agronomic nitrogen use efficiency (ANUE) of the individual treatments was calculated using the following equation as described previously (37).

$$\text{ANUE}=({\text{Y}}_{\text{N}}-{\text{Y}}_{0})/\text{N}$$

where N is the amount of nitrogen fertilizer applied (g N pot^−1^), Y_N_ is the crop yield (dry weight, g) with N applied, and Y_0_ is the crop yield (dry weight, g) in a control treatment without N applied. In this study, the amounts of the nitrogen fertilizers applied were 0.028 and 0.056 g pot^−1^ in the 50%CF and 100%CF treatments, respectively.

### Statistical analysis

The significant effects of various treatments were determined according to the magnitude of the F value (*P*=0.05). Treatment data were analyzed using an analysis of variance (ANOVA) followed by the Tukey-Kramer multiple comparison test.

### Sequence data

The accession numbers of the 16S rDNA, ITS, and *puf* gene sequences generated in this study were AB689796, AB823645, and AB828705 for the PS3 isolate; AB767253, AB823646, and AB828706 for the YSC3 isolate; and AB767254, AB823647, and AB828707 for the YSC4 isolate.

## Results

### Isolation and selection of PNSB isolates

**Fig. 1:**
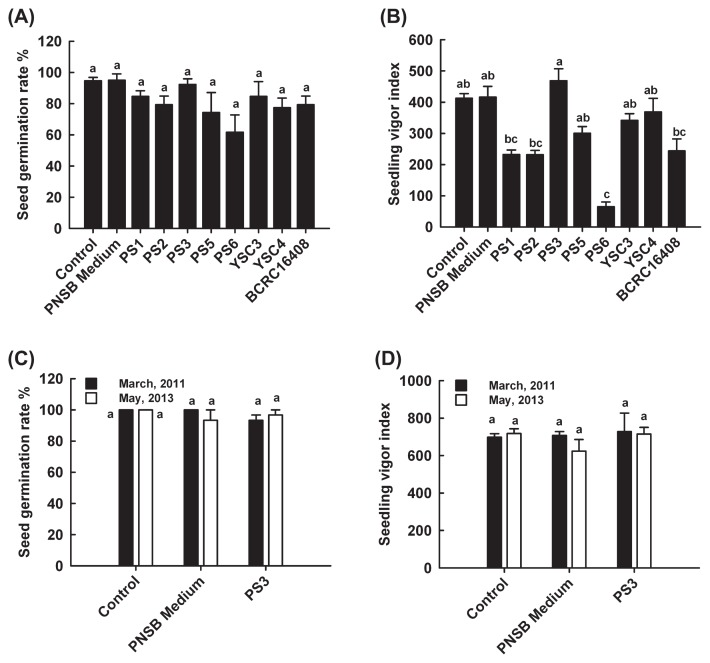
Effects of *R. palustris* inoculants on the seed germination rate (A) and seedling vigor index (B) of Chinese cabbage “Maruba Santoh” (*Brassica rapa* L. spp. *chinensis*). (C) The seed germination rate and (D) seedling vigor index in the old (March 2011) and new (May 2013) seeds treated with water (control), PNSB culture medium, or the PS3 inoculant. The data represent means ± SE, locations marked with different letters (a to c) are significantly different (*p* <0.05). Data are the means of three independent replicates. Percentage data were converted by the arcsine-square root transformation prior to analyses. Seeds of the control treatments were soaked in sterile water.

Two rhizosphere soil samples were screened for the presence of PNSB using a combination of the classical Winogradsky column method and molecular marker (*pufM* gene) detection. Seven of the PNSB isolates were pre-evaluated for their potential to promote plant growth by determining the resulting seed germination and seedling vigor (Fig. 1A and B). All of the isolates showed the same germination quality in Chinese cabbage as the non-treated control. Significant differences in the seedling-vigor tests were observed among the isolates. The PS3 isolate showed the highest seedling vigor index, followed by the YSC4 and the YSC3 isolates. The remainders, including the BCRC16408^T^ type strain, had a significantly lower seedling vigor index than that of the nontreated control. Accordingly, the PS3, YSC3, and YSC4 isolates were selected for further experiments in this study.

### Molecular phylogenetic analysis based on 16S ribosomal RNA gene (16S rDNA), *puf* gene, and ITS region sequences

**Fig. 2:**
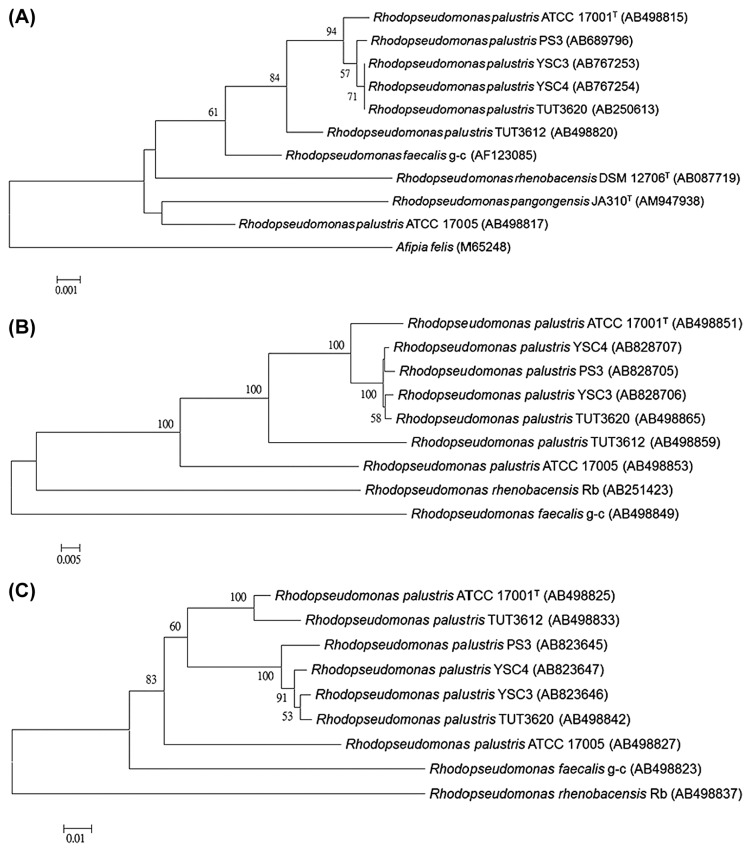
Phylogenetic tree based on 16S rDNA (A), *puf* gene (B), and 16S–23S rDNA ITS (C) sequences showing relationships between the genera *Rhodopseudomonas*. The trees were inferred by the NJ method. The accession numbers for the sequences are given in parentheses behind the strain names. Numbers in the nodes are the bootstrap values. The scale bar indicates that the number of substitutions per nucleotide position were 0.001 (A), 0.005 (B), and 0.01 (C). The sequence of *Afipia felis* was used as an outgroup to root in the 16S rDNA tree.

The 16S rDNA sequences of the three selected isolates (PS3, YSC3, and YSC4) were determined for their phylogenetic assignment. The isolates had nearly identical sequences (≥99.7%) with the *R. palustris* type strain ATCC17001^T^. A phylogenetic tree of the three isolates was constructed based on the 16S rDNA gene sequences of the *Rhodopseudomonas* strains retrieved from the NCBI database (Fig. 2a). All of them clustered with the *R. palustris* type strain ATCC17001^T^ and *R. palustris* TUT3620 strain, which was isolated from a hot spring. A phylogenetic analysis was conducted based on the *puf* gene. The *puf* genes, consisting of *pufL* and *pufM* genes encoding the two core proteins (L and M subunits) of the reaction center, have been used as markers in phylogenetic analysis of the genus *Rhodopseudomonas* (46). The sequences of the *puf* gene from the three isolates showed high similarities to each other (99%–100%). As shown in Fig. 2B, PS3 and YSC4 were clustered together and separated from YSC3 with a high bootstrap value (100%). The 16S-23S rDNA gene internal transcribed spacer (ITS) regions have been used to discriminate the phylogenetic relationships between closely related species (23). To obtain greater resolution among the closely related *R. palustris* isolates, we conducted a phylogenetic analysis based on the ITS regions. As shown in Fig. 2C, although the three isolates still belonged to the same cluster (bootstrap support of 100%), the three isolates could be further separated from each other into three clades (bootstrap support of 91%) by the neighbor-joining method.

### Physiological and biochemical characterizations of bacterial isolates

The colony and cell morphologies of the three *R. palustris* isolates and BCRC16408^T^ type strain were observed by optical microscopy and are shown in Fig. S1. The colonies that developed on agar medium under aerobic growth were pearlwhite, round, convex, smooth, and shiny, with entire edges (Fig. S1A). The colonies turned blood red when grown anaerobically under illumination (Fig. S1B). The cells were Gram-negative and had motile, rod-shaped cells approximately 1.0 μm in length (Fig. S1C). The physiological characteristics of the bacterial isolates are listed in Table 2. All three isolates were mesophilic, with a temperature growth range of 25–37°C and optimal growth at 30°C. The pH growth range of the three isolates was 5.0–9.0 (optimum pH 7.0), whereas the BCRC16408^T^ type strain grew at pH 6.0–8.0. NaCl was not obligatory for the growth of the isolates. The PS3 isolate could tolerate up to 1.5% w/v NaCl, whereas the other three strains tolerated up to 0.7% NaCl. As indicated by Meyer *et al.* (45), phototrophic bacteria exhibit light-dependent nitrogenase activity. The nitrogen-fixing ability of the isolates was measured using the ARA assay. The maximum nitrogenase activity was observed under microaerobic conditions in the light (Table 2). The PS3 isolate demonstrated the highest activity (8377.26 nmol C_2_H_4_ h^−1^ OD_600_^−1^ culture^−1^) (containing 10^7^ CFU mL^−1^), followed by the YSC4 and YSC3 isolates, while the BCRC16408^T^ strain had the lowest activity. In addition, all *R. palustris* isolates were able to produce indole-3-acetic acid (IAA), and isolate production ranged from 138.8 to 208.4 μM OD_600_^−1^. While performing the carbon source utilization assay for the isolates with the API 50CH commercial kit, no color change was observed in any test strip even after one week of incubation (data not shown). A positive test generally corresponds to the catabolism of substrates to produce acidic end products that can be detected using a pH indicator (phenol red). Phenol red is yellow at pH < 6.8 and red at pH > 7.4 (initial color), whereas pH = 6.8~7.4 is orange. We measured pH for the fermentation of carbohydrates by the *R. palustris* isolates, and found that the value was more than 7.4. To resolve this problem, we developed an API 50CH+LR method to evaluate the metabolism of carbohydrates by the *R. palustris* isolates. As shown in Table 2, PS3 utilized 16 types, YSC3 utilized 18 types, YSC4 utilized 8 types, and BCRC16408^T^ utilized 10 types of carbohydrate sources. Among the carbon sources, glycerol, D-glucose, D-fructose, salicin, amidon, D-arabitol, and potassium 5-ketogluconate were assimilated by all three of the isolates. A number of the carbohydrates gave strain-dependent results. For example, D-xylose and D-lyxose were only utilized by the PS3 isolate, and inulin, potassium gluconate, and potassium 2-ketogluconate were only utilized by the YSC3 isolate.

We determined the extracellular enzyme profiles of the isolates using the API ZYM system. All of the isolates were positive for C4 esterase, C8 esterase lipase, leucine aminopeptidase, acid phosphatase, and phosphoamidase (Table 2). Only the PS3 exhibited weak trypsin activity; YSC4 and BCRC16408^T^ exhibited β-glucosidase activity that was absent in PS3 and YSC3.

### Plant growth-promoting effects of bacterial treatments on Chinese cabbage

**Fig. 3:**
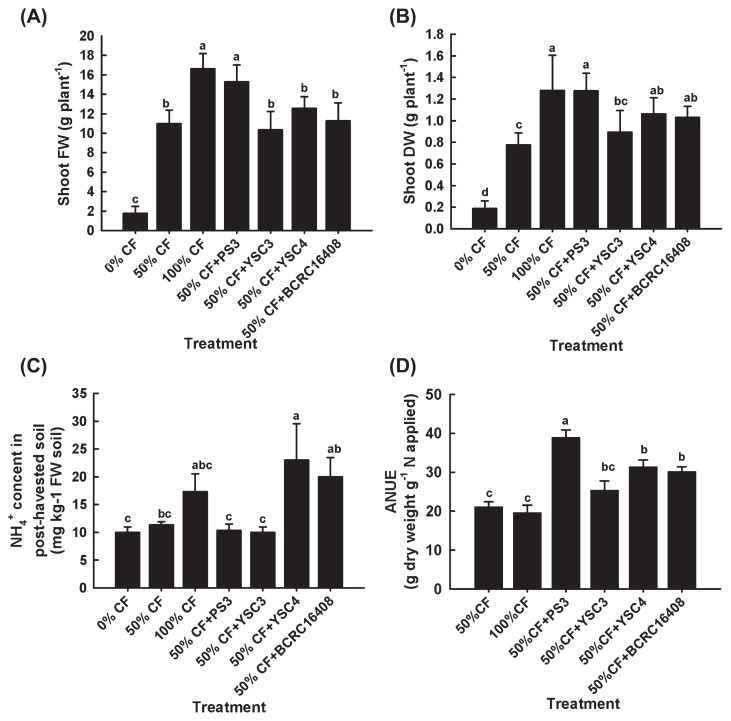
Plant growth-promoting effects of bacterial treatments on Chinese cabbage. (A) Fresh weights of shoots; (B) dry weights of shoots; (C) remaining amount of NH_4_^+^ ions in post-harvested soils; (D) agronomic nitrogen use efficiency (ANUE). The data represent means ± SE, locations marked with different letters (a to d) are significantly different (*p* <0.05). CF: chemical fertilizer, 50% CF+ respective *R. palustris* inoculant, inoculation of the bacterial strain (PS3, YSC3, YSC4, or the type strain BCRC16408^T^) with a half rate of fertilizer. The seeds used in the experiments were produced in March 2011.

We found that while inoculation with the *R. palustris* isolate (YSC3, YSC4 or PS3) on Chinese cabbage without chemical fertilizer application, none of them had an effect on plant growth (Fig. S2). To evaluate whether a reduction in the chemical fertilizer to 50% supplemented with the *R. palustris* isolates could promote the growth of Chinese cabbage similar to that achieved with full rates of fertilizer (100% CF) without inoculants, 1.2 × 10^9^ CFU of the respective inoculant was applied to each pot (equivalent to 4.0×10^6^ CFU g^−1^ soil). As shown in Fig. 3, the 100% CF promoted plant growth (fresh/dry weight of shoot) that was greater than that with the half amount (50% CF) of fertilizer alone. When 50% CF was supplemented with the PS3 inoculant, growth was significantly greater than that with the 50% CF treatment alone, and it was similar to that with 100% CF in both the fresh weights (Fig. 3A) and dry weights (Fig. 3B) of the shoots. When 50% of the fertilizer was supplemented with YSC3 or YSC4, the fresh weight of the shoots was lower than that with the 100% CF control (Fig. 3A), whereas no significant difference was observed in shoot dry weight between the YSC4 and 100% CF treatments (Fig. 3B).

We assessed the remaining N in the post-harvested soils using the Reflectoquant system. As shown in Fig. 3C, the concentration of NH_4_^+^ that remained in the soil following the 50% CF+PS3 or 50% CF+YSC3 treatments was lower than that remaining following the 100% CF treatment. On the other hand, the concentration of NO_3_^−^ was too low to be detected in all the tests (the limit of detection was 3 mg L^−1^). We further evaluated the agronomic nitrogen use efficiency (ANUE) of the individual treatments by calculating the increased dry yield per unit of the nitrogen fertilizer applied. As shown in Fig. 3D, the ANUE was higher with the *R. palustris* inoculation than with only chemical fertilization (either 50% or 100% CF), and that of the 50% CF+PS3 was superior to the other treatments.

**Fig. 4:**
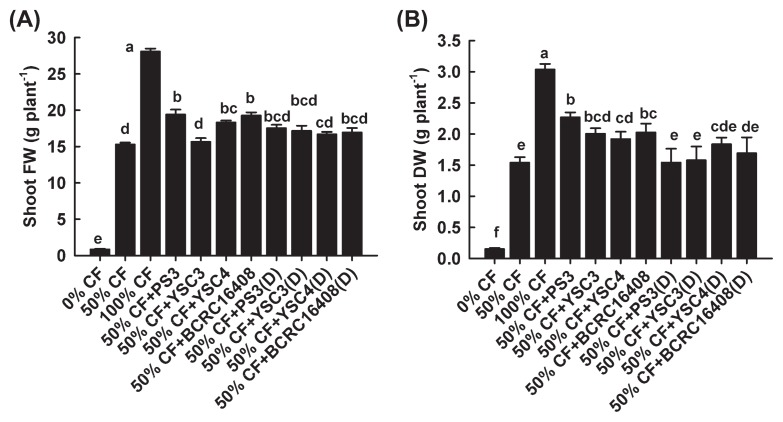
Effects of inoculation cell viability on plant growth by Chinese cabbage. (A) Fresh weights of shoots; (B) dry weights of shoots. The data represent means ± SE, locations marked with different letters (a to f) are significantly different (*p* <0.05). CF: chemical fertilizer, 50% CF+ respective *R. palustris* inoculant, inoculation of the bacterial strain (PS3, YSC3, YSC4, or the type strain BCRC16408^T^) with a half rate of fertilizer. D: 65°C heat-killed cells. The seeds used in the experiments were produced in May 2013.

We also conducted plant growth-promotion experiments using newly purchased seeds (produced in May 2013) (Fig. 4). As shown in Fig. 4A and B, the average fresh/dry weights of the plant shoots in the 50% CF and 50% CF+PS3 treatments were 15.30 ± 0.24/1.54 ± 0.04 g and 19.42 ± 0.67/2.26 ± 0.04 g, respectively. These results indicated that the growth of Chinese cabbage germinated from the new seeds under a half quantity of fertilizer was significantly increased by supplementation with the PS3 inoculant (up to 26.9% [FW] and 47% [DW]), and this inoculant was more effective than the other inoculants. However, growth was not similar to that of the 100% CF control.

To examine whether the beneficial effects of the *R. palustris* inoculants were elicited by viable cells or con- ferred by organic compounds from the medium or dead/decaying cells, a 65°C heat-killed bacterial suspension was applied to replace the vegetative *R. palustris* cells in the respective treatments. As shown in Fig. 4A and B, no significant difference was observed in either fresh or dry weight data between the 50%CF treatment and 50%CF+dead inoculant (PS3, YSC3, or BCRC16408^T^). This result indicated that the beneficial effects observed were mainly offered by viable cells.

### Survival of *R. palustris* inoculants in rhizosphere soils

To evaluate the survival rate of the inoculants, we analyzed the population of the respective *R. palustris* after 4 weeks of cultivation using the most probable number (MPN) method. The final bacterial concentrations were equivalent to 6.4×10^4^–5.7×10^5^ MPN g^−1^ soil for the 0% CF soils with inoculants, and 3.6×10^5^–5.7×10^6^ MPN g^−1^ soil for the 50% CF soils with inoculants (Table 3).

## Discussion

The *R. palustris* YSC3, YSC4 and PS3 isolates were originally isolated from Taiwanese rice paddy fields, and selected for pot trials based on their higher seedling vigor indexes (Fig. 1B). We demonstrated that inoculations with the isolates alone without applying any fertilizer failed to sustain normal plant growth (Fig. S2). To fulfill the requirement to reduce the conventional fertilizer dosage by at least half, we applied an integrated fertilization approach to search for promising PGPR inoculants from the *R. palustris* candidates. As shown in Fig. 3, all *R. palustris* isolates generally had beneficial effects on plant growth; however, only the PS3 strain had a marked impact on growth. We noted that although the original input of chemical fertilizer as well as the amount of remaining soil N were at the same level between the 50% CF and 50% CF+PS3 treatments (Fig. 3C), plant growth by the latter was significantly greater than that by the former, and similar to that of the 100% CF treatment (Fig. 3A and B). Moreover, the agronomic nitrogen use efficiency (ANUE) of the applied fertilizer nutrients was significantly enhanced following the PS3 treatment (Fig. 3D). ANUE is a parameter that reflects the ability of the plant to increase yield in response to the application of N (37). Accordingly, our results suggest that the application of N with the PS3 inoculant may have enhanced fertilizer efficiency in plants to supplement the nutrient requirements for crop growth at half the recommended rate of chemical fertilizer. Few studies have investigated agricultural applications of the bacterial species *R. palustris* alone on the growth of plants. As described above, Harada *et al.* (26) previously applied the *R. palustris* KN122 inoculant to promote the growth and grain yield of rice with a reduced amount of fertilizer. Taken together, these results suggest that *R. palustris* with careful selection may serve as potential single-strain inoculants for integrated nutrient management in either dry land or waterlogged cropping systems; however, additional field inoculation studies should be performed to verify these beneficial effects.

In this study, the dosage of the respective *R. palustris* inoculant applied to each pot was estimated to be 4.0×10^6^ CFU g^−1^ soil. The level of PGPR inoculants generally varies worldwide, and the dosage range is between 10^4^ and 10^9^ CFU g^−1^ soil (7, 8). Therefore, the amount of the inoculation used in this study was not markedly greater than those reported in the literature. In most cases, the populations of many PGPR inoculants declines progressively shortly after the bacteria are introduced into the soil (11). For example, Smith *et al.* (60) reported that the population of the widely used nitrogen fixing inoculant, *Azospirillum brasilense*, markedly decreased from 10^7^ to 10^2^ MPN g^−1^ of soil six weeks after the inoculation; the population of *Pseudomonas fluorescens* decreased from 10^9^ to 10^7^ CFU g^−1^ of soil after 29 d and to 10^4^ CFU g^−1^ of soil 4 weeks after the inoculation (66). As shown in Table 3, the survival of the *R. palustris* inoculants added to soil was more than 10^5^ MPN g^−1^ (3.6×10^5^–5.7×10^6^, except for the BCRC16408^T^ strain) after 4 weeks of cultivation. This result indicated that *R. palustris* isolates have a better survival rate than many PGPR reported previously. The survival strategies of the *R. palustris* strains remains to be elucidated on dry land because they were originally isolated from waterlogged paddy soils, and this bacterium is mainly distributed in aquatic ecosystems (29, 48, 52). Taken together, the strongly surviving populations of the *R. palustris* inoculants suggest that they can, by their persistence in soil, sustain their beneficial effects on plants during cultivation.

We noted that the plant-growth potential of Chinese cabbage was different between the old seeds (Fig. 3) and new seeds (Fig. 4). When pot trials were conducted with seedlings germinated from the old and new seeds, the shoot dry weights of the 100% CF plants were 1.28 ± 0.33 g (Fig. 3B) and 3.03 ± 0.04 g (Fig. 4B), respectively. This pattern suggests that the crop yield was highly affected by the quality of the seeds. As reported previously, seed quality influences seedling establishment, crop growth, and productivity (19). Therefore, farmers or seed providers employ the seedling vigor test to determine the physiological quality of seeds. Such tests are especially important for seeds under unknown or unfavorable storage conditions (30, 44). However, in this study, the seed germination rate (Fig. 1C) and seedling vigor (Fig. 1D) were hardly distinguishable between the two lots of Chinese cabbage seeds treated with water, the PNSB culture medium, or PS3 inoculant. It is possible that the deterioration of the old seeds was not obvious during the incubation period. On the other hand, we noted that the plant growth-promotion rate elicited by the PS3 inoculant at 50% fertility was markedly greater with the old seeds than with the new seeds. The former rate ranged up to 64% (Fig. 3B) while the latter showed a 47% (Fig. 4B) increase in the shoot dry weight over the control plants grown with 50% CF. These results suggest that the *R. palustris* PS3 inoculant can effectively boost the development of seedlings, particularly those germinated from carry-over seeds (*i.e.*, after storage, not fresh seeds) of poor quality, thereby improving plant growth and final crop yield to avoid costly damage.

The *R. palustris* PS3 isolate could produce IAA *in vitro* (approximately 140 μM OD_600_^−1^) (Table 2). The production of phytohormones has typically been regarded as the main factor affecting plant growth (14). However, PS3 was not the most outstanding IAA producer among the three isolates, indicating that the production of phytohormones may be not the main mode of action of PS3 when promoting plant growth. The PS3 isolate was superior for nitrogen fixation (8,400 nmol C_2_H_4_ h^−1^ OD_600_^−1^, as determined by ARA, Table 2) when cultivated anaerobically under light. Adesemoye *et al.* (4) indicated that biological nitrogen fixation not only directly supplied the essential nutrient to plants, but was also involved in the enhanced N uptake efficiency of inoculated plants. As described above, while treating soil with the PS3 inoculant with a half rate of chemical fertilizer, the nitrogen use efficiency of the applied fertilizer nutrients was significantly enhanced (Fig. 3D).

There is increasing evidence to suggest that plant root exudates play a key role in plant-microbe interactions (6, 20). The root exudate components, including carbohydrates, proteins, amino acids, and organic acids have been identified in different plant species and compiled in a review (15). According to the carbon assimilation profiles shown in Table 2, the PS3 isolate has the potential to utilize a greater variety of plant-derived carbohydrates (for example, L-arabinose, D-ribose, D-xylose, D-glucose, and D-fructose) than other isolates. Since carbon fluxes are considered to be crucial determinants of rhizosphere function (63), it is critical that future research focuses on the spatial and temporal dynamics of carbohydrates between plants and PS3 cells.

Plant-growth stimulation by PGPR has been regarded as the net result of multiple mechanisms that may be activated simultaneously (43). Taken together, we propose that the PS3 inoculant exerts its beneficial effects on plants through combined modes of actions, including the synthesis of phytohormones (IAA), improvements in nutrient metabolism by plants, along with its high colonization and persistence abilities, as described above. Further analyses of e genetic and metabolic profiles in response to plant root exudates can provide a comprehensive understanding of the underlying PGP mechanisms elicited by *R. palustris* species.

## Supplementary Information



## Figures and Tables

**Table 1:** Primers used in this study

Amplified genes	Sequence (5′→3′)	Amplicon (bp)	Reference
16S rDNA		1450	Eden *et al.*, 1991 (18)
Eub 27F	GAGTTTGATCCTGGCTCAG		
Univ 1492R	GGTTACCTTGTTACGACTT		
16S–23S ITS		973–1043	Okamura *et al.*, 2009 (49)
SS 1512F	GTCGTAACAAGGTAGCCGT		
LS 117R	GGGTTTCCCCATTCGGAAATC		
*puf*		1528	Nagashima *et al.*, 1997 (46)
pufL	CTGTTCGATTTYTGGGTYGG		
pufM	CCCATSGTCCAGCGCCAGAA		
*puf*M		229	Achenbach *et al.*, 2001 (2)
pufM557F	CGCACCTGGACTGGAC		
pufM750R	CCCATGGTCCAGCGCCAGAA		

Mixtures of bases used at certain positions are given as: S = G or C; Y = C or T

**Table 2:** Phenotypic characteristics of PS3, YSC3, YSC4, and *R. palustris* (Molish) van Niel BCRC16408 strains

Characteristic	PS3	YSC3	YSC4	*R. palustris*
Origin	rice paddy soil, Taipei City, Taiwan	rice paddy soil, Yilan County, Taiwan	rice paddy soil, Yilan County, Taiwan	BCRC16408^T^ (ATCC17001^T^)
Temperature range for growth (°C)	25–37	25–37	25–37	25–37
pH range for growth	5~9	5~9	5~9	6~8
Salinity range for growth (%)	0–0.7	0–1.5	0–0.7	0–0.7
Gram staining	G-	G-	G-	G-
Nitrogenase activities^a^ (nmolC_2_H_4_/h/OD_600_)	8377.3 ± 22.1	55.3 ± 6.7	922.9 ± 45.8	25.3 ± 1.5
IAA production (μM/OD_600_)^b^	138.8 ± 9.8	169.2 ± 8.5	190.5 ± 11.0	208.4 ± 41.3
Carbohydrate source utilization				
glycerol	+	+	+	+
L-arabinose	+	+	−	−
D-ribose	+	+	−	−
D-xylose	+	−	−	−
D-adonitol	−	+	−	+
D-glucose	+	+	+	+
D-fructose	+	+	+	+
L-sorbose	+	+	−	−
inositol	−	−	−	+
amygdain	+	+	−	−
esculin; ferric citrate	+	+	−	−
salicin	+	+	+	+
inulin	−	+	−	−
D-raffinose	−	−	−	−
amidon	+	+	+	+
D-turanose	+	+	−	−
D-lyxose	+	−	−	−
D-tagatose	+	+	−	+
D-arabitol	+	+	+	−
L-arabitol	−	−	+	+
potassium gluconate	−	+	−	−
potassium 2-ketogluconate	−	+	−	−
potassium 5-ketogluconate	+	+	+	+
API ZYM tests^c^				
C-4 esterase	4	3	3	5
C-8 esterase lipase	4	4	4	3
leucine aminopeptidase	5	4	4	3
cystine aminopeptidase	1	1	0	0
trypsin	1	0	0	0
acid phosphatase	2	1	2	2
phosphoamidase	2	1	2	2
ß-glucosidase	0	0	2	2

a and b Data represent means ± SD (standard deviation)

c The API ZYM test values ranging from 0–5: 0 corresponds to a negative reaction, 5 to a reaction of maximum intensity and values 1, 2, 3, or 4 are intermediate reactions depending on the level of intensity

**Table 3:** Survival of *R. palustris* inoculants in rhizosphere soils after 4 weeks of cultivation

	Final bacterial concentration Log (MPN g^−1^ soil)^a^
0% CF+P3	5.41
0% CF+YSC3	5.76
0% CF+YSC4	4.96
0% CF+BCRC16408	4.80
50% CF+PS3	6.18
50% CF+YSC3	6.76
50% CF+YSC4	5.80
50% CF+BCRC16408	5.56

a Data represent the means of two independent replicates.

CF: chemical fertilizer, the dosage for the respective inoculation was approximately 4.0 × 10^6^ CFU g^−1^ soil.

## References

[1] Abdul-Baki AA, Anderson JD (1973). Vigor determination in soybean seed by multiple criteria. Crop Sci.

[2] Achenbach LA, Carey J, Madigan MT (2001). Photosynthetic and phylogenetic primers for detection of anoxygenic phototrophs in natural environments. Appl Environ Microbiol.

[3] Adesemoye AO, Torbert HA, Kloepper JW (2008). Enhanced plant nutrient use efficiency with PGPR and AMF in an integrated nutrient management system. Can J Microbiol.

[4] Adesemoye AO, Torbert HA, Kloepper JW (2009). Plant growth-promoting rhizobacteria allow reduced application rates of chemical fertilizers. Microb Ecol.

[5] Altschul SF, Madden TL, Schaffer AA, Zhang J, Zhang Z, Miller W, Lipman DJ (1997). Gapped BLAST and PSI-BLAST: a new generation of protein database search programs. Nucleic Acids Res.

[6] Bais HP, Weir TL, Perry LG, Gilroy S, Vivanco JM (2006). The role of root exudates in rhizosphere interactions with plants and other organisms. Annu Rev Plant Biol.

[7] Bashan Y, Puente ME, Rodriguez-Mendoza MN, Toledo G, Holguin G, Ferrera-Cerrato R, Pedrin S (1995). Survival of *Azospirillum brasilense* in the bulk soil and rhizosphere of 23 soil types. Appl Environ Microbiol.

[8] Bashan Y (1998). Inoculants of plant growth promoting rhizobacteria for use in agriculture. Biotechnol Adv.

[9] Berg G (2009). Plant-microbe interactions promoting plant growth and health: perspectives for controlled use of microorganisms in agriculture. Appl Microbiol Biotechnol.

[10] Bhattacharyya PN, Jha DK (2012). Plant growth-promoting rhizobacteria (PGPR): emergence in agriculture. World J Microbiol Biotechnol.

[11] Brockwell J, Gault RR, Chase DL, Hely FW, Zorin M, Corbin EJ (1980). Appraisal of practical alternatives to legume seed inoculation—field experiments on seed bed inoculation with solid and liquid lnoculants. Aust J Agric Res.

[12] Carlozzi P, Sacchi A (2001). Biomass production and studies on *Rhodopseudomonas palustris* grown in an outdoor, temperature controlled, underwater tubular photobioreactor. J Biotechnol.

[13] Carpenter SR (2005). Eutrophication of aquatic ecosystems: bistability and soil phosphorus. Proc Natl Acad Sci USA.

[14] Costacurta A, Vanderleyden J (1995). Synthesis of phytohormones by plant-associated bacteria. Crit Rev Microbiol.

[15] Dakora FD, Phillips DA (2002). Root exudates as mediators of mineral acquisition in low-nutrient environments. Plant Soil.

[16] Diaz RJ, Rosenberg R (2008). Spreading dead zones and consequences for marine ecosystems. Science.

[17] Dubey RC, Maheshwari DK, Maheshwari DK (2011). Role of PGPR in integrated nutrient management of oil seed crops. Bacteria in Agrobiology: Plant Nutrient Management.

[18] Eden P, Schmidt T, Blakemore R, Pace N (1991). Phylogenetic analysis of *aquaspirillum magnetotacticum* using polymerase chain reaction-amplified 16S rRNA-specific DNA. Int J Syst Evol Microbiol.

[19] Ellis R (1992). Seed and seedling vigour in relation to crop growth and yield. Plant Growth Regul.

[20] Fan B, Carvalhais L, Becker A, Fedoseyenko D, von Wiren N, Borriss R (2012). Transcriptomic profiling of *Bacillus amyloliquefaciens* FZB42 in response to maize root exudates. BMC Microbiol.

[21] Fawzy ZF, El-magd AMM, Li Y, Ouyang Z, Hoda AM (2012). Influence of foliar application by EM, “effective microorganisms”, amino acids and yeast on growth, yield and quality of two cultivars of onion plants under newly reclaimed soil. J Agric Sci.

[22] Felsenstein J (1985). Confidence limits on phylogenies: an approach using the bootstrap. Evolution.

[23] García-Martínez J, Acinas SG, Antón AI, Rodríguez-Valera F (1999). Use of the 16S–23S ribosomal genes spacer region in studies of prokaryotic diversity. J. Microbiol Methods.

[24] Grahamweiss L, Bennett ML, Paau AS (1987). Production of bacterial inoculants by direct fermentation on nutrient-supplemented vermiculite. Appl Environ Microbiol.

[25] Harada N, Nishiyama M, Matsumoto S (2001). Inhibition of methanogens increases photo-dependent nitrogenase activities in anoxic paddy soil amended with rice straw. FEMS Microbiol Ecol.

[26] Harada N, Nishiyama M, Otsuka S, Matsumoto S (2005). Effects of inoculation of phototrophic purple bacteria on grain yield of rice and nitrogenase activity of paddy soil in a pot experiment. Soil Sci Plant Nutr.

[27] Harrison FH, Harwood CS (2005). The pimFABCDE operon from *Rhodopseudomonas palustris* mediates dicarboxylic acid degradation and participates in anaerobic benzoate degradation. Microbiology.

[28] Harwood CS, Gibson J (1988). Anaerobic and aerobic metabolism of diverse aromatic compounds by the photosynthetic bacterium *Rhodopseudomonas palustris*. Appl Environ Microbiol.

[29] Hiraishi A, Kitamura H (1984). Distribution of phototrophic purple nonsulfur bacteria in activated sludge systems and other aquatic environments. Bull Jap Soc Sci Fish.

[30] Hungria M, Loureiro MF, Mendes IC, Campo RJ, Graham PH, Werner D, Newton W (2005). Inoculant preparation, production and application. Nitrogen Fixation in Agriculture, Forestry, Ecology, and the Environment.

[31] Hunter CN, Daldal F, Thurnauer MC, Beatty JT (2008). The purple phototrophic bacteria.

[32] Imhoff JF, Dworkin M, Falkow S, Rosenberg E, Schleifer KH, Stackebrandt E (2006). The phototrophic alphaproteobacteria. The Prokaryotes: A Handbook on the Biology of Bacteria Proteobacteria: Alpha and Beta Subclass.

[33] Javaid A (2006). Foliar application of effective microorganisms on pea as an alternative fertilizer. Agron Sustain Dev.

[34] Karpinets TV, Pelletier DA, Pan C, Uberbacher EC, Melnichenko GV, Hettich RL, Samatova NF (2009). Phenotype fingerprinting suggests the involvement of single-genotype consortia in degradation of aromatic compounds by *Rhodopseudomonas palustris*. PLoS ONE.

[35] Kloepper JW, Leong J, Teintze M, Schroth MN (1980). Enhanced plant growth by siderophores produced by plant growth-promoting rhizobacteria. Nature.

[36] Kumar S, Pandey P, Maheshwari D (2009). Reduction in dose of chemical fertilizers and growth enhancement of sesame *Sesamum indicum* L. with application of rhizospheric competent *Pseudomonas aeruginosa* LES4. Eur J Soil Biol.

[37] Ladha JK, Pathak H, Krupnik TJ, Six J, van Kessel C (2005). Efficiency of fertilizer nitrogen in cereal production: Retrospects and prospects. Adv Agron.

[38] Larimer FW, Chain P, Hauser L (2004). Complete genome sequence of the metabolically versatile photosynthetic bacterium *Rhodopseudomonas palustris*. Nat Biotechnol.

[39] Lian S, Wang C, Lee Y, Morris R (1996). Analysis of fertilizer responses and efficiencies of fertilizers applied to vegetables in the Hsilo area of Taiwa. Managing Soil Fertility for Intensive Vegetable Production Systems in Asia.

[40] Lin M (2002). Fertilizer Use by Crop in Taiwan.

[41] Lucy M, Reed E, Glick BR (2004). Applications of free living plant growth-promoting rhizobacteria. Antonie Van Leeuwenhoek.

[42] Lugtenberg B, Kamilova F (2009). Plant-Growth-Promoting Rhizobacteria. Annu Rev Microbiol.

[43] Martínez-Viveros O, Jorquera MA, Crowley DE, Gajardo G, Mora ML (2010). Mechanisms and practical considerations involved in plant growth by Rhizobacteria. J Soil Sci Plant Nutr.

[44] McDonald MB, Copeland LO (1997). Seed Production: Principles and Practices.

[45] Meyer J, Kelley B, Vignais P (1978). Effect of light nitrogenase function and synthesis in *Rhodopseudomonas capsulata*. J Bacteriol.

[46] Nagashima KV, Hiraishi A, Shimada K, Matsuura K (1997). Horizontal transfer of genes coding for the photosynthetic reaction centers of purple bacteria. J Mol Evol.

[47] Nakkeeran S, Fernando W, Siddiqui Z, Siddiqui Z (2006). Plant growth promoting rhizobacteria formulations and its scope in commercialization for the management of pests and diseases. PGPR: Biocontrol and Biofertilization.

[48] Oda Y, Wanders W, Huisman LA, Meijer WG, Gottschal JC, Forney LJ (2002). Genotypic and Phenotypic Diversity within Species of Purple Nonsulfur Bacteria Isolated from Aquatic Sediments. Appl Environ Microbiol.

[49] Okamura K, Takata K, Hiraishi A (2009). Intrageneric relationships of members of the genus *Rhodopseudomonas*. J Gen Appl Microbiol.

[50] Patten CL, Glick BR (2002). Role of *Pseudomonas putida* indoleacetic acid in development of the host plant root system. Appl Environ Microbiol.

[51] Rana A, Saharan B, Joshi M, Prasanna R, Kumar K, Nain L (2011). Identification of multi-trait PGPR isolates and evaluating their potential as inoculants for wheat. Ann Microbiol.

[52] Roper MM, Ladha JK (1995). Biological nitrogen fixation by heterotrophic and phototrophic bacteria in association with straw. Plant Soil.

[53] Saharan BS, Nehra V (2011). Plant growth promoting rhizobacteria: a critical review. Life Sci. Med. Res.

[54] Saitou N, Nei M (1987). The neighbor-joining method: a new method for reconstructing phylogenetic trees. Mol Biol Evol.

[55] Sarker SD, Nahar L, Kumarasamy Y (2007). Microtitre plate-based antibacterial assay incorporating resazurin as an indicator of cell growth, and its application in the *in vitro* antibacterial screening of phytochemicals. Methods.

[56] Sasikala C, Ramana CV (1998). Biodegradation and metabolism of unusual carbon compounds by anoxygenic phototrophic bacteria. Adv Microb Physiol.

[57] Schmidhalter U (2005). Development of a quick on-farm test to determine nitrate levels in soil. J Plant Nutr Soil Sci.

[58] Shaharoona B, Naveed M, Arshad M, Zahir ZA (2008). Fertilizer-dependent efficiency of Pseudomonads for improving growth, yield, and nutrient use efficiency of wheat (*Triticum aestivum* L.). Appl Microbiol Biotechnol.

[59] Singh JS, Pandey VC, Singh DP (2011). Efficient soil microorganisms: a new dimension for sustainable agriculture and environmental development. Agric Ecosyst Environ.

[60] Smith RL, Schank S, Milam J, Baltensperger A (1984). Responses of *Sorghum* and *Pennisetum* species to the N2-fixing bacterium *Azospirillum brasilense*. Appl Environ Microbiol.

[61] Suzuki S, Aono T, Lee K-B, Suzuki T, Liu C-T, Miwa H, Wakao S, Iki T, Oyaizu H (2007). Rhizobial factors required for stem nodule maturation and maintenance in *Sesbania rostrata- Azorhizobium caulinodans* ORS571 symbiosis. Appl Environ Microbiol.

[62] Thompson JD, Higgins DG, Gibson TJ (1994). CLUSTALW: improving the sensitivity of progressive multiple sequence apalignment through sequence weighting, positions-specific gap penalties and weight matrix choice. Nucleic Acids Res.

[63] Toal M, Yeomans C, Killham K, Meharg A (2000). A review of rhizosphere carbon flow modelling. Plant Soil.

[64] van Niel CB, Anthony San P (1971). Techniques for the enrichment, isolation, and maintenance of the photosynthetic bacteria. Methods Enzymol.

[65] Vessey JK (2003). Plant growth promoting rhizobacteria as biofertilizers. Plant Soil.

[66] Wessendorf J, Lingens F (1989). Effect of culture and soil conditions on survival of *Pseudomonas fluorescens* R1 in soil. Appl Microbiol Biotechnol.

[67] Yamada K, Xu H-L (2001). Properties and applications of an organic fertilizer inoculated with effective microorganisms. J Crop Prod.

