# Supplementary material for: Promoting Effects of a Single Rhodopseudomonas palustris Inoculant on Plant Growth by Brassica rapa chinensis under Low Fertilizer Input
Source: Microbes Environ. 2014 Aug 12;29(3):303–13. doi: 10.1264/jsme2.ME14056 (PMC4159042; doi:10.1264/jsme2.ME14056)
Supplement: Supplementary file 1 [file 29_303_s1.pdf]

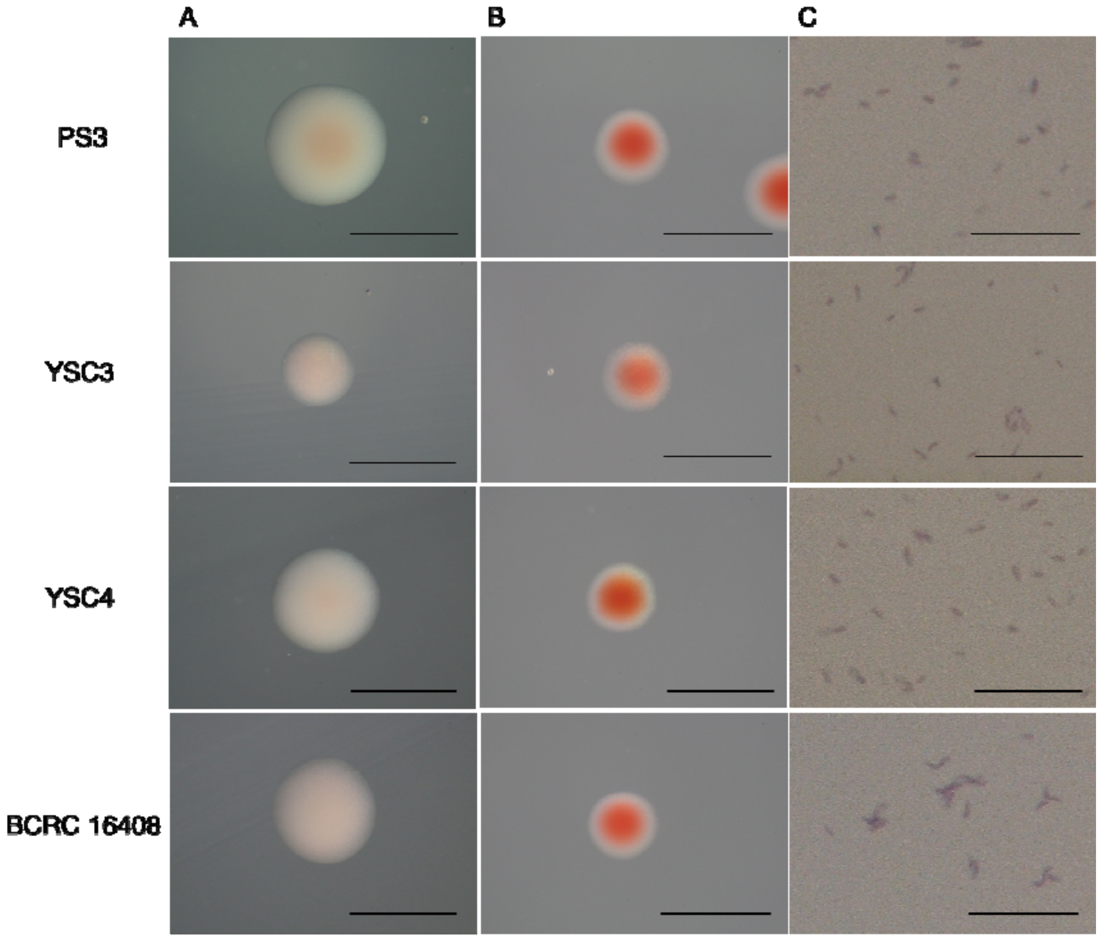

Fig. **S1** Morphological characteristics of PS3, YSC3 and YSC4 isolates and *R. palustris* BCRC16408 type strain. **(A)** Colonies developed under aerobic growth (4 days); **(B)** colonies grown anaerobically under illumination (7 days); **(C)** vegetative cells cultivated aerobically at 30°C in PNSB broth. Scale bars equal 0.5 cm in panels **A** and **B**, 10 µm in panel **C**.

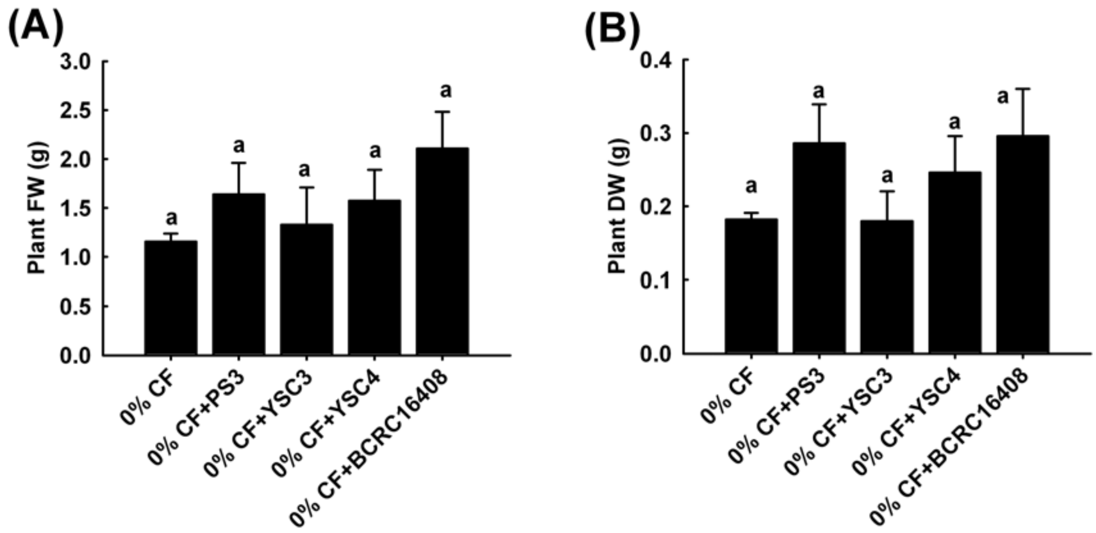

Fig S2. Effects of the *R. palustris* inoculants alone on plant growth of Chinese cabbage at 0%CF. (**A**) Fresh weight of plant; (**B**) dry weight of plant. CF: chemical fertilizer, 0% CF+ respective *R. palustris* inoculant, inoculation of the bacterial strain (PS3, YSC3, YSC4, or the type strain BCRC16408) without fertilizer application. The data represent means ± SE.
